# COVID-19 Research: Pandemic *Versus* “Paperdemic”, Integrity, Values and Risks of the “Speed Science”

**DOI:** 10.1080/20961790.2020.1767754

**Published:** 2020-06-10

**Authors:** Ricardo Jorge Dinis-Oliveira

**Affiliations:** Department of Public Health and Forensic Sciences, and Medical Education, Faculty of Medicine, University of Porto, Porto, Portugal; Department of Sciences, IINFACTS-Institute of Research and Advanced Training in Health Sciences and Technologies, University Institute of Health Sciences (IUCS), CESPU, CRL, Gandra, Portugal; UCIBIO-REQUIMTE, Laboratory of Toxicology, Department of Biological Sciences, Faculty of Pharmacy, University of Porto, Porto, Portugal

**Keywords:** SARS-CoV-2, COVID-19, research and academic integrity, peer review, pandemic, paperdemic

## Abstract

Scientific integrity is a learned skill. When researchers and students learn integrity in laboratories or in the classroom, they are empowered to use similar principles in other aspects of their lives. This commentary reviews the concepts related to scientific integrity at a time when science faces important challenges related to the increase number of articles produced regarding research on coronavirus disease 2019 (COVID-19). Severe acute respiratory syndrome coronavirus 2 (SARS-CoV-2) has ignited another parallel viral pandemic, with science ranging from robust studies to dishonest studies being conducted, posted, and shared at an unprecedented rate. A balance is needed between the benefits of the rapid access to new scientific data and the threat of causing panic or erroneous clinical decisions based on mistakes or misconduct. The truth is that the “scientific research has changed the world” but now, and more than ever, “it needs to change itself”. A pandemic with a “paperdemic” will be even more complicated to manage if it progresses in an uncontrolled manner and is not properly scrutinized.

## Introduction

In late December 2019, an outbreak of an emerging disease (coronavirus disease 2019, COVID-19) due to a novel virus named severe acute respiratory syndrome coronavirus 2 (SARS-CoV-2) started in Wuhan, China, and rapidly spread throughout the world [1]. The World Health Organization (WHO) declared the epidemic of COVID-19 as a pandemic on 12 March 2020. Since then, research on SARS-CoV-2 became unique in this context. Indeed, articles related to SARS-CoV-2 are published every minute in high-impact journals, thus demonstrating how popular the topic currently is [2]. To obtain an estimate of the scale of research activity, *Nature* found approximately 900 papers, preprints and preliminary reports published prior to 12 March when searching for English studies using the terms “novel coronavirus”, “ncov”, “COVID-19” and “SARS-CoV-2” on the bioRxiv, medRxiv, ChemRxiv and ChinaXiv servers, as well as compiling publications listed by the WHO and on Google Scholar [2]. Since Chinese-language journals were excluded, the number is obviously underestimated.

Nevertheless, the article’s content in several cases, under normal circumstances, would be published in predatory publications and the time between submission and acceptance in several articles is much less than a week in several articles. Therefore, it is obvious that the peer review process is being weakened and was never so rife in coronavirus papers [3].

Of course, these are difficult times and we need rapid results to save people, and some journals are “asking editors to accept without delay submitted manuscripts that in their judgment can stand as eLife papers, even if they feel that the manuscript would be stronger” [4]. Although this is an altruistic attitude aiming fast publication without delay, and prone to some degree of success, the truth is also that accepting everything is not helping medicine, quite the opposite. Indeed, reducing the rigor of studies would not lead to any benefits, may distort COVID-19 knowledge, and postpone a real solution for pandemic. Therefore, it is important that scientific journals remain firm and focused on assuring quality. But in these critical moments, scientific journals that have high-impact factors and are indexed in the best databases of medicine, should not deprive researchers and physicians of credible research. Scientific journals are the last strongholds we have for evidence-based medicine, about the care of individual patients [5].

In the context of COVID-19, preprint servers are also a study case with unprecedented use of these platforms [6]. They allow that the scientific information, unrestricted by text limits or demands for complete articles, to be communicated, read, and scrutinized almost immediately. While there is widespread agreement that preprints can be useful, there are significant risks associated with such an unregulated process due to the spread of faulty information into the public domain without third-party screening. Indeed, never the history of science was produced so much non-peer-reviewed data and not only by preprint servers but also by traditional publications. In another point of view, what seemed like a failure of preprints, sometimes is a success since the widespread dissemination can also help to rapidly detect intentional and unintentional error, and blocks poor quality-research. The erroneously claiming that COVID-19 contained human immunodeficiency virus (HIV) “insertions” was one of the first retracted preprints, in this case withdrawn by authors [7]. More problematic is the fact that with the speed of their release, preprints “rather than peer-reviewed literature in the same topic area, might be driving discourse related to the ongoing COVID-19 outbreak” [6].

We should also not forget that many researchers and academics acknowledge that they have already engaged in fraudulent behaviour [8], leading to substantial economic and social costs [9]. In addition to the direct financial waste resulting from academic and research misconduct, the negative implications are much broader for society. Indeed, scientific studies often induce changes in medical interventions, such as in the case of vaccinations [10, 11]. The consequences of rushed publication are also notorious when *Cell* accepted a controversial cloning-related article, from the scientific and ethical point of view, in just 3 days [12]. The prevalent culture of “publications at any cost” and “publish or perish” appears to be responsible for this distrust [13].

This commentary highlights the importance of scientific and academic integrity during the SARS-CoV-2 crisis and aims to alert health professionals, especially doctors and forensic experts, that they should carefully read scientific articles and not blindly trust the findings, even if the journal has a high impact factor or is untouchable from a scientific point of view, as these articles are not all applicable. It is mandatory that researchers and decision makers, including politicians, act responsibly to ensure the scientific validity of studies conducted during outbreaks as well as the participants’ rights and safety in these studies.

## Concepts of scientific and academic integrity

Scientific and academic integrity are mutually dependent, and especially in biomedical literature, it is particularly important in ensuring science-based clinical and forensic best practices [14, 15]. As previously mentioned, the protection and promotion of academic and research integrity is the responsibility of several actors, including individual researchers, peers, funding bodies, regulatory bodies, journals, namely editorial boards, publishers, and institutions that employ research staff and administer research activities [14, 15]. Regarding **scientific integrity,** there is currently no universal definition but implies adherence to ethical and professional principles, values and practices when conducting and applying the results of science and scholarship [16]. Besides ensuring objectivity, honesty, clarity, reproducibility and utility, scientific integrity is also important since it provides insulation from the following: bias that would aim to privilege some lines of investigation or results over others, fabrication, falsification, plagiarism, and conflicts of interest. The European Code of Conduct for Research Integrity Revised Edition [17,18], highlights the fundamental principles/values for research in searching for the truth: i) honesty in developing, undertaking, reviewing, reporting and communicating research in a transparent, fair, full and unbiased way; ii) reliability in ensuring the quality of research, reflected in the design, the methodology, the analysis and the use of resources; iii) respect for colleagues, research, participants, society, ecosystems, cultural heritage and the environment; and iv) accountability for the research from idea to publication, for its management and organization, for training, supervision and mentoring, and for its wider impacts. The Scientific Integrity Consortium [16] developed a set of recommended principles and best practices that can be used broadly across scientific disciplines as a mechanism for consensus on scientific integrity standards and to better endow scientists to operate in a rapidly changing research environment. The two principles that represent the umbrella under which scientific processes should operate are as follows: i) foster a culture of integrity in the scientific process; and ii) evidence-based policy interests may have legitimate roles to play in influencing aspects of the research process, but those roles should not interfere with scientific integrity. The nine best practices for instilling scientific integrity in the implementation of these two overarching principles were also postulated.

Regarding **academic integrity**, the International Centre for Academic Integrity (ICAI) provides a forum to identify, affirm, and promote the values of academic integrity among students, faculty, teachers, and administrators. The ICAI defines academic integrity as a commitment to five fundamental values, which are consistent with the scientific research values highlighted above: honesty, trust, fairness, respect, and responsibility [19]. The ICAI states that these five values, as well as the courage to act on them even in the face of adversity, are truly foundational to the academy [19].

## Scientific misconduct and other unethical practices

If we consider that the intrinsic goal of science is the will of truth, we must admit that it has recently suffered serious blows that threaten its integrity, with several medical researchers reporting misconduct more frequently than respondents in other fields [20]. Several reports of academic and scientific fraud have been disclosed in scientific publications, such as *Nature*, *Science* or *Lancet*, and on several electronic sites, such as Retraction Watch (https://retractionwatch.com/) and PubPeer (https://pubpeer.com/). Particularly, PubPeer allows users to anonymously discuss and review scientific research, allowing a post-publication peer review. Nevertheless, the number of cases of misconduct that have come to public knowledge will probably represent only “the tip of the iceberg” [21]. Traditional media have also reported these situations, leading to a high impact on public opinion. This phenomenon is not new, but it has become more visible and less tolerated in recent years [22]. A detailed review of all 2 047 biomedical and life-science research articles indexed as retracted by PubMed on May 3, 2012 revealed that only 21.3% of retractions were attributable to error. In contrast, 67.4% of retractions were attributable to misconduct, including fraud or suspected fraud (43.4%), duplicate publication (14.2%), and plagiarism (9.8%) [21]. For instance, from 1996 to 2013, Yoshihiro Sato, a Japanese bone-health researcher, committed plagiarism, fabricated data and authorship, resulting in the retraction of more than 60 scientific studies, including clinical trials [23]. He allegedly committed suicide, but this fact has yet to be confirmed [23]. In this case, it was clear that journals, publishers, and institutions had slowly undertaken misconduct investigations.

### Types of research misconduct

The term “scientific fraud”, which was used in the literature as a distinctive label of all these situations, has, due to the imposition of a legal regime, been gradually replaced by the term “scientific misconduct”. The basis of this conceptual change is the normative framework of the “fraud” regime. Indeed, for instance in American law, “fraud” presupposes the verification of proof and the demonstration of dishonesty and damage caused to a certain victim. However, in most cases of scientific research, the requirement of an existing victim is not suitable to fulfil the concept of “fraud”. Therefore, in several countries, both terms are used interchangeably [18, 24]. According to the thesaurus dictionary, it “represents an ethically unacceptable breaking of confidence perpetrated for profit or to gain some unfair or dishonest advantage”. In other words, it is also a deliberately/intentional false representation of the truth. It has been typically classified using the so-called “cardinal sins” FFP categorization, meaning falsification, fabrication and plagiarism (Table 1) [25, 26]. Although all are treated in the same category as a moral offense, plagiarism is somewhat situated on a different level of offense because it is an offense to the scientific community but not to the truth of science [18, 27]. Moreover, self-plagiarism is typically not considered research misconduct [17].

**Table 1:** Subtypes of research misconduct [25, 26].

Subtypes	Description
Fabrication	The making up of data or results and the recording or reporting them as if they were real
Falsification	The manipulation of research materials, equipment or processes, or the change or omission of data or results without justification such that the research is not accurately represented in the research record
Plagiarism	The term is derived from the Latin word *plagiārius*, meaning “kidnapper” or “abductor”, and *plagium*, “kidnapping”. The appropriation of another person’s published and unpublished ideas, processes, results or words (or other intellectual property) without giving appropriate credit to the original source and thus violating the rights of the original author(s) to their intellectual outputs. The intent and effect of plagiarism is to mislead the reader as to the contributions of the plagiarizer. The USA Office of Research Integrity defines plagiarism as being “theft or misappropriation of intellectual property and the substantial unattributed textual copying of another's work”

### Other forms of violations of good research practice

The three forms of violation described above are considered particularly serious since they distort the research record. Nevertheless, there are additional violations of research integrity (Table 2). Although they are less valued and visible, they are probably more frequent and should not be ignored since they also damage the integrity of the research process or of researchers [28]. These other forms also present different degrees of gravity, and the prevalence of each of them is also unknown.

**Table 2:** Other forms of violations of a good research practice besides misconduct.

No.	Violation forms
1	Fraudulent identification of some authors
2	Not fulfilling the conditions for an authorship (e.g., authorship offer)
3	Manipulating authorship or devaluing the role of other researchers in publications, for instance, by the inaccurate position of an author in article
4	Failure to cite relevant work by other researchers: the authors claim that they are the first to describe a finding while neglecting to note a similar contribution previously published by other authors
5	Purchase and sale of articles
6	Supervision of dozens of MSc and PhD students at the same time
7	Omitting conflicts of interest between authors during peer review or with the pharmaceutical industry and other funders or sponsors
8	Not acknowledging those who deserve acknowledgments
9	Publishing in predatory journals
10	Self-plagiarism: re-publishing substantive parts of one’s own earlier publications, including translations, without duly acknowledging or citing the original
11	Redundant publication: risk of doing an investigation that has already been done by neglecting the search for previous bibliography on the subject
12	Misuse of other didactic material
13	Citing selectively (i.e., to increase the h-index) or to please editors, reviewers or coworkers
14	Withholding research results and attempting to publish incomplete investigations
15	Unnecessarily expanding the bibliography of a study
16	Conducting human studies without informed consent
17	Accusing a researcher of misconduct or other violations in a malicious way
18	Misrepresenting research achievements
19	Exaggerating the importance and practical applicability of findings
20	Inadequate protection of the people and animals participating in research
21	Fake peer review

Although it does not affect the quality of the research or impact the value of the produced knowledge, the false coauthorship of an article (i.e. if the intellectual contribution for the developed work is limited) affects the careers of researchers and the reputation of the research institution [29]. The classic case is the “Department Director”, who demands to be a coauthor based only on hierarchical status. To prevent such violations, many journals have requested a declaration of coauthorship, with an explanation of what each author contributed to the article. Moreover, if the contribution of each author is clear, the position in which his/her name appears would not matter. According to the International Committee of Medical Journal Editors [29], authorship credits should be based only on substantial contributions to: i) the conception, planning, analysis or interpretation of data; ii) the writing of the article or its critical review; iii) the responsibility for the final approval for publication; and iv) the assumption of responsibility for the results and the integrity of any part of the work. All four conditions must be fulfilled for an authorship, and the contribution of those who do not meet the criteria listed above should be listed, with their permission, in the acknowledgements section.

At a different level, we can still find “bad”, but honest science, due to methodological or other types of errors, inadequate reagents, misinterpreted data, errors in the tests, negligence, or ethically objectionable behavior [20, 21].

## Possible causes of research misconduct

Regarding the causes of research misconduct, several have been reported in the literature, and they are typically grouped into two types: structural/organizational misconduct and individual misconduct [20, 30, 31].

From the structural/organizational point of view, the main causes that threaten scientific ethics include the lack of ethically robust and fair institutional policies focusing on scientific integrity, the inadequate supervision and tutoring of students, the assessment of the researcher’s prestige and the scientific impact of his/her work, the “publish or perish” aphorism, and the absence of skills for interpreting bibliometric variables such as the impact factor and h-index [32–36]. Scientific output and academic progression should not be assessed only based on the granted budget for projects, self-funding through grants, bibliometric indicators, as such assessments may constitute a serious threat to scientific robustness [37, 38]. While bibliometrics is a highly useful science, it should not be the unique assessment [39]. In other words, it is important to redefine excellence [40] and to base the evaluation of a researcher not only on metrics but also on fair judgement [41]. Moreover, academic institutions play important roles in protecting and preserving research integrity. Recently, Grey et al. [15] evaluated the objectivity, adequacy and transparency of institutional investigations of potentially compromised research integrity and identified important deficiencies in the quality and reporting of institutional investigation involving their own research or academic staff.

Regarding individual concerns, since science is a major human activity, there will always be individuals who exhibit deviant behaviours and engage in research misconduct [24]. Individuals with the following traits are claimed to be more susceptible to engage in misconduct: a socially maladjusted personality; vanity; a desire to achieve a positive scientific reputation and recognition by peers; and a passionate belief in a particular theory, line of research or scientific thesis that sometimes converts the researcher in a non-open/non-flexible mind for other possibilities [42, 43]. Nevertheless, it also true that all these traits of personality can also be, if appropriately applied, useful to achieve honest results. Pathological publishing is being recognized as a new psychological disorder with legal consequences and was interestingly reviewed by Buela-Casal [44]. The author proposal is based on the fact that several criteria that suggest maladaptation, are clearly met: falsification and/or manipulation of data, falsification of publication indicators, distortion of reality, belief in manipulated data, and an obsession with conducting marketing campaigns for oneself. In an interesting study [45], an association between a creative personality and mindset, and dishonesty and unethical behaviour, was found.

## Possible consequences of research misconduct

Science has an ethical basis that must be cultivated. Scientific misconduct has impacts for researchers, research participants, institutions, and society [24]. Among the most obvious consequences are those related to the reduced reproducibility of clinical research, the direct and indirect financial costs associated with scientific misconduct, and the legal and judicial outcomes. For example, in cancer research, fraud-based results lead to highly reduced reproducibility, which has obvious negative impacts on patients treated on the basis of such results [46–48]. Moreover, retracting an article that presents the results of a clinical study may take several months or years [49–55].

Another aspect that deserves reflection is the scientific supervision of multiple master’s or doctoral students among some researchers. Whether supervisors are forced into this situation or they want it, they often act as supermen and end up in an unrealistic situation. In such cases, students are not adequately supervised, and a culture of ethics and good scientific conduct is not created. Moreover, unfair competition for budgets or prestige is also created, since a greater number of PhD students leads to higher productivity but inevitably less scrutiny. For instance, the number of science doctorates earned each year grew by nearly 40% between 1998 and 2008 in countries that are members of the Organization for Economic Co-operation and Development (OECD), reaching a total of approximately 34 000 degrees [56]. Therefore, doctoral education and the functional definition of the doctorate remain a matter of debate throughout the world, with several additional demands, activities, responsibilities, duties, and opportunities. This represents new challenges aiming professionalize all stakeholders of academic and research institutions responsible for doctoral training. As suggested by other authors [57], this evolution also involves a resignification of the conception of the doctorate itself and the nomenclature that we give to the stakeholders involved, namely, supervisors and PhD students. A distant supervisor with intellectual superiority and authority focused only on research output needs to be replaced by a professional who, in addition to being a researcher, is someone who can simultaneously take on the roles of expert, mentor, coach, manager, evaluation and professional counsellor [58]. To achieve these goals, institutions should not consider supervisors as supermen and should develop efforts aiming to professionalize doctoral supervision in a global and international context. On the other hand, PhD students should be considered PhD researchers or candidates. Finally, the relationship between both agents should be a “professional researcher-training researcher” relationship rather than a “teacher-student” relationship [57].

Regarding financial waste, a study examining publications retracted due to serious misconduct estimated that the direct cost to the National Institutes of Health (NIH – USA) was approximately US$4 25 000 per article [59]. Another study focusing on the costs of research misconduct calculated that the direct cost to the institution was approximately US$5 00 000 and that the total cost of all reported claims was approximately US$11 00 00 000 [60]. Ranjit Kumar Chandra, who was accused of fraud and financial deception by the Canadian Broadcasting Corporation (CBC), has been ordered to pay the broadcaster just over C$1.6 million in cost after losing a court case [61]. Gammon and Franzini [9], by using sequential mixed methods, found that research misconduct among faculty at academic medical centers could cause a cost ranging from US$1 16 160 to $21 92 620 per case.

Court trials for research misconduct rarely occur. Cases within institutions are typically addressed by contract cessation, the exposure of what happened publicly, the expiration of a license for professional practice or the cancelation or nonrenewal of a budget for research. Such infractions can ruin the professional career of the researcher, the group of researchers, coauthors and the institution itself. The impact also exists for whistleblowers. Often, they are themselves victims of negative consequences in their personal and professional life as well as discomfort in the work environment, forcing them to have to look for a job elsewhere, even if their attitude is virtuous and ethical [62].

Forensic reports and consequently judicial decisions can also be affected if they are based on scientific data produced in the context of misconduct or if scientific articles are not properly scrutinized. Deserves to be mentioned, in the negative, a recent publication in forensic field, without any sense of meaning. The speculation regarding contamination with COVID-19 during autopsy was postulated [63]. Although we could not exclude this risk, since an open mind to all possibilities is part of the researcher spirit, the raised “observations” in this publication did not justify conclusions. Deserves also to be mentioned, in the positive, the subsequent publication, where the same authors, with good faith “regret that the article might not have good writing for clarification and it might result in misinterpretation” [64]. In a post-publication peer review evaluation, it seems to me that the greatest author’s difficulty was language limitations, as sometimes occurs in many non-native English speakers, as me. However, it looks obvious that the pre-publication peer review, even being a *Letter to Editor*, unsuccessfully demonstrated the scientific background of the article.

On the other hand, forensic experts can also help science to analyze laboratory research records and to uncover misconduct [65–68]. Therefore, proper procedures for investigating allegations of research misconduct, by including skilled forensic experts, would be particularly useful.

## Scientific policies and proposals for resolutions

Several countries have long understood the importance and relevance of examining scientific misconduct. Accordingly, they have created policies and structures that specifically address this problem, which has not yet happened in the case of Portugal and in several other countries. It is therefore important to implement an integrated strategy in different vectors, which are listed below.

### Education

Aiming to promote scientific and academic integrity, all players involved must attend specific educational programs and research groups and institutions must foster the creation of an ecosystem, in which leaders demonstrate concern and attention with integrity and full lack of condescension regarding misconduct. It is also important to have well-defined procedures that guide the investigation of suspected research misconduct in all institutions [15]. Moreover, a dedicated committee to address research misconduct should be part of the permanent structure of an institution. To produce reliable conclusions, those committees should act independently and with transparency in the investigation of suspected cases, assuring the confidentiality of collected data and proposing actions to the institutions’ executive and disciplinary bodies. Even when investigations are exemplary and findings clear, universities rarely report them publicly and therefore it was recently proposed to make reports of research misconduct public [69]. In a post pandemic Era, with a more clear and open mind, we will have enough time to scrutinize several fast publications, and it would be an important scientific contribution and advance to have journals and media once more together in helping researchers to internalize valid scientific knowledge for the future.

### National organization

At the national level, it is essential to create a governmental organization that includes representatives of different public and private higher academic institutions that can coordinate preventive actions and act in suspected cases of academic and scientific misconduct for all areas of knowledge. This “National Agency for Scientific and Academic Integrity” must have the power and the means to investigate those cases, as is the case in some European countries such as Denmark and Norway [70] and in the United States of America (i.e. The Office of Research Integrity) since the 1980s. This agency should also be based on an interministerial initiative, based on Ministries of Science, Technology and Higher Education and Justice. Among other functions, this agency could produce guidelines aiming to stimulate scientific research in accordance with the values of integrity discussed above. For further inspiration, the following documents offer relevant insights [17, 71, 72].

## Analyses of specific publications for COVID-19 treatment

COVID-19-related research is an excellent example of how responsible leadership and researchers should behave with respect to news of potential new treatments. At this moment, considering the current scientific knowledge, several drugs are under investigation, such as remdesivir, which is used for the treatment of Ebola virus; lopinavir-ritonavir, which is used for the treatment of immunodeficiency virus (HIV); favipiravir for the treatment of influenza virus; and chloroquine or hydroxychloroquine, which is used for malaria, but most data are no more than anecdotal evidence. Although, it is not clear that misconduct was practiced, since at this time the principle of *in dubio pro reo* needs to be respected, it is obvious that “speed science” has become very troubling with torrent of bad science and with lot of peers commenting on the lack of reliability of several results. Indeed, the ongoing fast peer review process in COVID-19-related research, is potentially damaging science and compromises the research integrity principles, even without misconduct. In the following topics, some examples of therapeutics in the modern medicine claimed to be useful for COVID-19 [73] are discussed and, when applicable, the perspective that results were rapidly contradicted is presented. In some cases, it will be made clear the hurried results, the speculations, and a considerable lack of scientific foundations. Indeed, while before pandemic several months were invested from submission to publication, COVID-19 shortened the time to few days in several cases, increasing the changes of producing unsound results.

### Azithromycin and hydroxychloroquine

The effectiveness of the combination of azithromycin and hydroxychloroquine (i.e. a derivative of chloroquine) for the treatment of COVID-19 is far from being clarified. Although it is not implausible that these drugs could inhibit viral replication in humans, the scientific polemic of this therapeutics probably started with the publication of Didier Raoult as correspond author [74]. Authors observed that hydroxychloroquine treatment was significantly associated with viral load reduction/disappearance in COVID-19 patients and its effect was reinforced by azithromycin. In the conclusion of this study, the authors “recommended that COVID-19 patients be treated with hydroxychloroquine and azithromycin to cure their infection and to limit the transmission of the virus to other people in order to curb the spread of COVID-19 in the world” [74, 75]. Nevertheless, this open-label non-randomized clinical trial had some very glaring methodological flaws, such as a very small sample, a lack of randomization, ethical problems, missing patients, confounding variables, and few inclusion and exclusion criteria [74]. Many others have also been reported by the PubPeer Journal Club and Retraction Watch. At least one needs to be detailed since the “speed science” was in its best with an incredible ultrafast peer review. Indeed, besides the authors, mention of a fact dated from 14 March, the article was submitted on 16 March, accepted on 17 March and published online on 20 March. Given the urgency of the COVID-19 crisis, some limitations of this study may be acceptable, but it is becoming obvious that peer review failed in providing a prudent article. Previously, hydroxychloroquine has been demonstrated to exhibit anti-SARS-CoV activity *in vitro* [76]. Wang et al. [77] also demonstrated *in vitro* that chloroquine was highly effective in the control of infection.

It was most probably the publication of Didier Raoult as correspond author [74] that triggered USA and several other countries to give emergency approval to hydroxychloroquine despite a lack of complete evidence [78]. Food and Drug Administration (FDA) former agency executives claimed that “the emergency use authorization for two malaria drugs to treat COVID-19, based on thin evidence of efficacy, has jeopardized research to learn the drugs’ real value against the pandemic coronavirus” [79]. Moreover, this therapeutic had an important politician ally. Indeed, the President Donald Trump, acting as a medical adviser, said that “hydroxychloroquine and azithromycin, taken together, have a real chance to be one of the biggest game changers in the history of medicine”. After that, the President Donald Trump has repeatedly returned to this claim and lead to an Emergency Use Authorization (EUA) to buy at least 29 million doses of hydroxychloroquine for national supply.

Indeed, no evidence of rapid antiviral clearance or clinical benefits due to the combination of hydroxychloroquine and azithromycin in patients with severe COVID-19 infection was subsequently reported [80]. Recently, a panel of experts of the National Institute of Allergy and Infectious Diseases recommends against doctors using a combination of hydroxychloroquine and azithromycin for the treatment of COVID-19 due to toxic effects of both drugs, such as QT interval prolongation. Moreover, in a preprint, mortality rate increased with hydroxychloroquine in veterans hospitalized with COVID-19. Authors in a weighted attitude, suggested that these “findings highlight the importance of awaiting the results of ongoing prospective, randomized, controlled studies before widespread adoption of these drugs” [81]. Besides the toxicity, another consequence is the increasingly inadequate supply of hydroxychloroquine for patients in whom efficacy is established, such as patients with rheumatoid arthritis and systemic lupus erythematosus [82]. Accordingly, it was recommended that “governments need to think twice before they suppress messages related to COVID-19” [83].

### Tocilizumab and ciclesonide

The complex role that the immune system might play in COVID-19 has been disclosed after the use of tocilizumab for the treatment of severe patients. Indeed, the pathogenesis of SARS related to coronavirus involves a cytokine storm with higher plasma levels of cytokines interleukin (IL)-6, IL-2, IL-7, IL-10, interferon gamma inducible protein (IP10), monocyte chemoattractant protein (MCP1), macrophage inflammatory protein (MIP1A) and tumour necrosis factor (TNF-α) [84]. Therefore, tocilizumab, an anti-IL6 receptor antibody, has been proposed for the treatment of COVID-19 [73, 85, 86]. Moreover, the inhaled corticosteroid ciclesonide apparently inhibits the coronavirus RNA replication by targeting viral NSP15 [87, 88].

### Ritonavir and lopinavir

HIV type 1 aspartate protease inhibitors have also been proposed for the treatment of COVID-19, but results are highly contradictory. Specifically, the oral efficacy and safety of ritonavir, combined with lopinavir to increase its plasma half-life through the inhibition of cytochrome P450, was evaluated in a randomized, controlled, open-label trial in adult patients hospitalized [89]. Results did not reveal additional benefit in comparison to the standard treatment. Indeed, the authors found that adding lopinavir-ritonavir treatment did not reduce viral RNA loads or the duration of viral RNA detectability compared with standard supportive care alone. Moreover, important adverse effects were registered, and hepatic injury, pancreatitis, severe cutaneous eruptions, QT prolongation, and the potential for multiple drug interactions due to CYP3A inhibition, are also well documented with this drug combination [89].

### Remdesivir

Remdesivir is a prodrug that is intracellularly metabolized to an analogue of adenosine triphosphate that inhibits viral RNA polymerases [77]. When remdesivir was provided on a compassionate-use basis to patients hospitalized with COVID-19, clinical improvement was observed in 36 of 53 patients [90]. Nevertheless, this is an uncontrolled study since other factors may have contributed to differences in outcomes, including the type of supportive care (e.g. concomitant medications or variations in ventilatory practices) and differences in institutional treatment protocols and thresholds for hospitalization. The authors concluded, that the assessment of the efficacy will require ongoing randomized, placebo-controlled trials of remdesivir therapy [90]. Optimistic comments demonstrating that patients responded promptly to remdesivir treatment, were also made public from a site investigator [91]. Additional encouraging signs of the clinical benefit of remdesivir in rhesus macaques infected with SARS-CoV-2 were published in a preprint server [92]. More recently, hospitalized patients with advanced COVID-19 and lung involvement who received remdesivir recovered faster than patients who received placebo, according to a preliminary data analysis from a randomized, controlled trial involving 1 063 patients [93]. Then FDA [94] granted emergency use in patients hospitalized with severe COVID-19 who require oxygen supplementation.

On the other hand, discouraging results of a partially completed study appeared for a short time on the WHO website [95]. The publication was then removed but a screen shot was made available.

### Ivermectin

Ivermectin, an approved anti-parasitic drug, is another suggested treatment [96]. It was shown to have broad-spectrum anti-viral activity against SARS-CoV-2 *in vitro*, with a single addition of 5 µmol/L to Vero-hSLAM cells 2 h post infection leading to an ∼5 000-fold reduction in viral RNA at 48 h. Interestingly, no toxicity of ivermectin was observed at any of the timepoints tested. Nevertheless, ivermectin should be further investigated for possible benefits in humans, specially the success of the highest currently approved doses (200 µg/kg) [96].

### Amiodarone

Amiodarone, an antiarrhythmic drug, also proved able to block the spreading of SARS-CoV infection in cell cultures probably due to interference with the endocytic pathway [97]. A concise review, highlighting amiodarone importance in the treatment of coronavirus infection, was recently published [98], but there is a very long way to prove its efficacy in COVID-19 treatment.

### Nicotine

Recent media news reported that researchers are suggesting that nicotine patches may be useful in COVID-19 patients, due to the preliminary observations that smokers may be much less at risk of being infected and have a very much lower probability of developing symptomatic or severe SARS-CoV-2 infection [99, 100]. Indeed, it has been hypothesized that the nicotinic acetylcholine receptor (nAChR) plays a key role in the pathophysiology of COVID-19 infection and might represent a target for the prevention and control infection [101].

### Melatonin

Finally, a brief mention for a review that summarizes the “likely benefits of melatonin in the attenuation of COVID-19” accordingly to the pathogenesis. This “speculation” is based on the well-known anti-inflammatory and anti-oxidative effects of melatonin, which are useful mechanisms to counteract the acute lung injury (ALI)/acute respiratory distress syndrome (ARDS) that may develop in COVID-19 patients. An original study is needed to demonstrate melatonin efficacy [102].

## The specific case of ibuprofen and COVID-19 progression

Ibuprofen is a non-steroidal anti-inflammatory drug (NSAIDs) widely used as antipyretic. The link between ibuprofen exposure and the increased risk of COVID-19 progression is probably one of the major confusions and problems of “speed science”, to not say more. Due to my particularly interest in toxicology and pharmacology, I have been frequently contacted by parents, researchers, physicians, pharmacists, and several other professionals to offer them further therapeutic clarification regarding this topic. The first report was made by the French Health Minister, the neurologist Olivier Véran, who said that “taking anti-inflammatory drugs (e.g. ibuprofen, cortisone) could be an aggravating factor for the infection. If you have a fever, take paracetamol” [103]. Since then, several health professionals and medias were discouraging ibuprofen use with a tremendous influence on clinical decisions but also on the public’s trust in science. But where is the science behind the publications on this subject?

Firstly, it was demonstrated that SARS-CoV-2 binds to its target cells through angiotensin-converting enzyme 2 (ACE2), which is expressed by epithelial cells of the lung, intestine, kidney, and blood vessels [104]. The expression of ACE2 is substantially increased in patients with type 1 or type 2 diabetes who are treated with ACE inhibitors and angiotensin II type-I receptor blockers (ARBs) and therefore, both classes could favour virus transmission. Based on these conclusions, it was recently claimed that ACE2 can also be increased by thiazolidinediones and ibuprofen [105]. The authors hypothesized that ibuprofen could increase the expression of ACE2 without any justification or scientific reference. This sentence had no scientific validity, and it was tremendously disturbing and alarming justifying the European Medicines Agency (EMA), WHO and several other health associations intervention to clarify healthcare practitioners and all society. Indeed, the publication was a commentary, which means that there is no medical evidence. Later, on 17 March [103], several researchers were advising that “ibuprofen should not be used for managing symptoms, according to doctors and scientists”. On 23 March, the same author [106] presented a different view, and the real value of these publications caused no more than noise and confusion. Previously, a single reported study demonstrated that the expression of ACE2 in cardiac tissue was found to be decreased by more than 65% in diabetic rats [107], but results were not further corroborated. Finally, EMA stated that when starting treatment for fever or pain in COVID-19, patients and healthcare professionals should consider all available treatment options, including paracetamol and NSAIDs.

## Conclusion and future perspectives

Now that everyone has realized that we have a real pandemic that could overwhelm hospitals with COVID-19 patients and kill millions of people worldwide, it is understandable that society is fighting and desperate for an effective vaccine or treatment. In these circumstances, the best contribution of scientists is to provide reliable scientific information through evidence-based medicine. In other words, it is mandatory that researchers and decision makers be deeply responsible in scientific research. Nevertheless, it is becoming obvious that the COVID-19 pandemic has resulted in a considerable number of scientific related articles, and the speed at which the information has been published is not compatible with the time classically spent during the scrutiny of peer reviews. Moreover, it is highly probable that as the pandemic progresses, we will see more preprints posted or more servers such as bioRxiv, medRxiv, ASAPbio and ChemRxiv publishing preliminary studies, and there will be several unsustainable results specially claiming efficient treatments. Specifically, while preprints are important for a scientific debate and obtaining input and feedback, some of them are too speculative and were widely shared on social media, further spreading the findings to the public, and thus causing fear and wrong decisions. It is commendable that the preprint server for Biology, namely, bioRxiv, and other servers have now added a yellow warning banner across the top of any new COVID-19 research that deserves to be transcribed: “bioRxiv is receiving many new papers on coronavirus 2019-nCoV. A reminder: these are preliminary reports that have not been peer-reviewed. They should not be regarded as conclusive, guide clinical practice/health-related behaviour, or be reported in news media as established information”.

This race for scientific publications is certainly based on the pressure to publish, with the aim of being the first to find a solution, leading to career advancement and promotions. Moreover, some researchers are taking advantage of the less careful performance of scientific journals during the COVID-19 crisis. The society will not benefit from early findings if they are weak and widely publicized. Additionally, these results could have a dramatic consequence since some of the healthcare policy responses to COVID-19 have been based on misleading, and at times incorrect, information. Therefore, risky decisions are being made even by the most qualified professionals who understand medical language, such as doctors.

Journals are also important players in assuring quality. In this concern, the reality has become fiction itself, and the other side of the coin should be considered. In the recent past, authors have benefited from predatory journals of low quality to publish their results. The high-quality scientific journals were the last bastion to guide credible knowledge and strengthen decision-making. In other words, these high-quality journals were useful for distinguishing wheat from chaff. Nevertheless, with “publication fever”, authors, editors and reviewers all lose the control of their function. It should not be forgotten that the Committee on Publication Ethics (COPE), a reputable organization whose recommendations are currently followed by quality scientific journals, has prepared guiding documents that the editors could follow during their activity, including guidelines on how to deal with scientific misconduct [108].

Finally, at the institutional level, ethical committees should apply the most rigorous standards to authorize research in accordance with the principles of justice, equity, and solidarity. Particularly, in 2016, the WHO published the “guidance for managing ethical issues in infectious disease” [109] to ensure the scientific validity of studies conducted during outbreaks as well as participants’ rights and safety in these studies. The climate of fear may predispose patients to agree to participate in research where experimental drugs are being used and inclusion and exclusion criteria are not being properly documented [110]. An interesting viewpoint aiming to assure clinical trial integrity during COVID-19 pandemic was recently presented [111]. Moreover, it is important for all healthcare practitioners to pay attention to all article retractions in order to adjust recommendations for diagnosis and treatments to be modified accordingly [112].

In conclusion, reading an interesting post from Reuteus Graphics and enclosed figures is suggested [113]. The authors highlighted that “while speedy scientific analysis is highly useful if it’s good, flawed or misleading science can sow panic and may make a disease epidemic worse by prompting false policy moves or encouraging risky behaviour”. The future will also disclose if those with scientific background are more capable of filtering and interpreting scientific results. That will probably highlight the importance and the competences of being a health or a forensic specialist with or without a regular contact with research. At least in my field, I believe that having forensic routine case work supported in regular research offers notorious advantages [114]. The importance of a scientific background was also recently suggested to be a key contributing factor to Germanic success in managing pandemic due to Angel Merkel doctorate in quantum chemistry [115]. Finally, the arguments anti pseudoscience are weakened if trusted medical institutions condemn an evidence-free practice in one context and legitimize it in another [116]. The publication rhythms are changing rapidly with COVID-19 and "some crazy claims and predictions about things that might treat" the disease are being published [117]. The fight against research misconduct and misinformation should be, more than ever, a researcher responsibility.
